# Acute Retinal Necrosis in an Immunocompetent Patient Treated With Intravitreal Ganciclovir

**DOI:** 10.7759/cureus.17816

**Published:** 2021-09-08

**Authors:** Mouhamed Nashawi, Tyler Bahr, Trent Palmer

**Affiliations:** 1 Department of Internal Medicine, Baylor Scott and White All Saints Medical Center, Fort Worth, USA; 2 Department of Ophthalmology, University of Texas Health Science Center, San Antonio, USA

**Keywords:** necrosis, retina, intravitreal, vision, ocular, fundus, intravitreal injections, acute retinal necrosis, antiviral

## Abstract

Acute retinal necrosis (ARN) is an inflammatory syndrome of high clinical concern; untreated or misdiagnosed cases may progress to optic neuropathy or retinal detachment, leading to irreversible blindness. ARN affects men and women equally and is often seen in immunocompromised patients but is also known to present in immunocompetent patients. It is usually due to systemic viral infection with secondary vitreoretinal inflammation. Most commonly, the first-line management of ARN is oral or intravenous antiviral therapy. Here, we report the case of an immunocompetent patient presenting with necrotizing retinopathy secondary to ARN. This patient was treated with oral valacyclovir and then intravenous acyclovir with no improvement. However, intravitreal injection of ganciclovir successfully halted the progression of ARN and led to the preservation of vision in the patient.

## Introduction

Acute retinal necrosis (ARN) is an inflammatory syndrome associated with the presence of peripheral retinal necrosis, occlusive vasculopathy, panuveitis including inflammation of the anterior chamber, and vitreous involvement. Although it is a rare disease with an estimated incidence of at least 0.63 cases per million per year, ARN is of clinical concern because untreated or misdiagnosed cases may progress to optic neuropathy or retinal detachment, leading to irreversible blindness [1]. Patients with ARN may present with unilateral acute vision loss, floaters, pain, and redness [2,3]. While ARN has a proclivity to affect one eye, it may present bilaterally in some instances [4,5]. Because these complaints are broad within the context of ophthalmologic pathologies, this makes delineation of the condition arduous to a clinician who is unfamiliar with or inexperienced in managing this entity. The nonspecific nature of symptoms, when weighted against the resultant risk of critical vision loss, warrants vigilance and consideration of an appropriate workup in patients who have a past medical, social, or surgical history that raises the index of suspicion for ARN.

The pathogenesis of ARN usually occurs in two phases - the acute phase involving inflammatory insult due to viral burden with subsequent infiltration of inflammatory cells and associated vessel occlusion, which over the course of a few weeks can progress to the late cicatricial phase which involves fibrotic traction on the retina and retinal detachment. Due to the rapid progression of ARN in the absence of treatment and the subsequent risk of permanent vision loss, it is imperative that antiviral therapy be started immediately. Furthermore, treatment with acyclovir has been shown to halt lesion progression within the first 48 hours of starting intravenous antiviral therapy. According to the literature, the causative viruses are primarily varicella-zoster virus (VZV), herpes simplex virus (HSV), cytomegalovirus (CMV), and Epstein-Barr virus (EBV) [6]. Viral subtyping in ARN is typically achieved through polymerase chain reaction (PCR) of ocular fluid to detect specific viral DNA sequences. Due to the viral etiology of this condition, standard treatment involves intravenous antivirals followed up with oral antiviral medication, while some complex cases (as described primarily in literature) further warrant intravitreal antiviral therapy [7-9]. Discrepancies in the literature exist on whether or not intravitreal therapy improves visual outcomes in patients with ARN when compared with oral or intravenous antiviral therapy [9,10]. We report the case of an immunocompetent patient presenting with necrotizing retinopathy secondary to ARN who did not improve with oral or intravenous antivirals. However, intravitreal injections halted the progression of ARN and led to the preservation of vision in the patient.

## Case presentation

**Figure 1:**
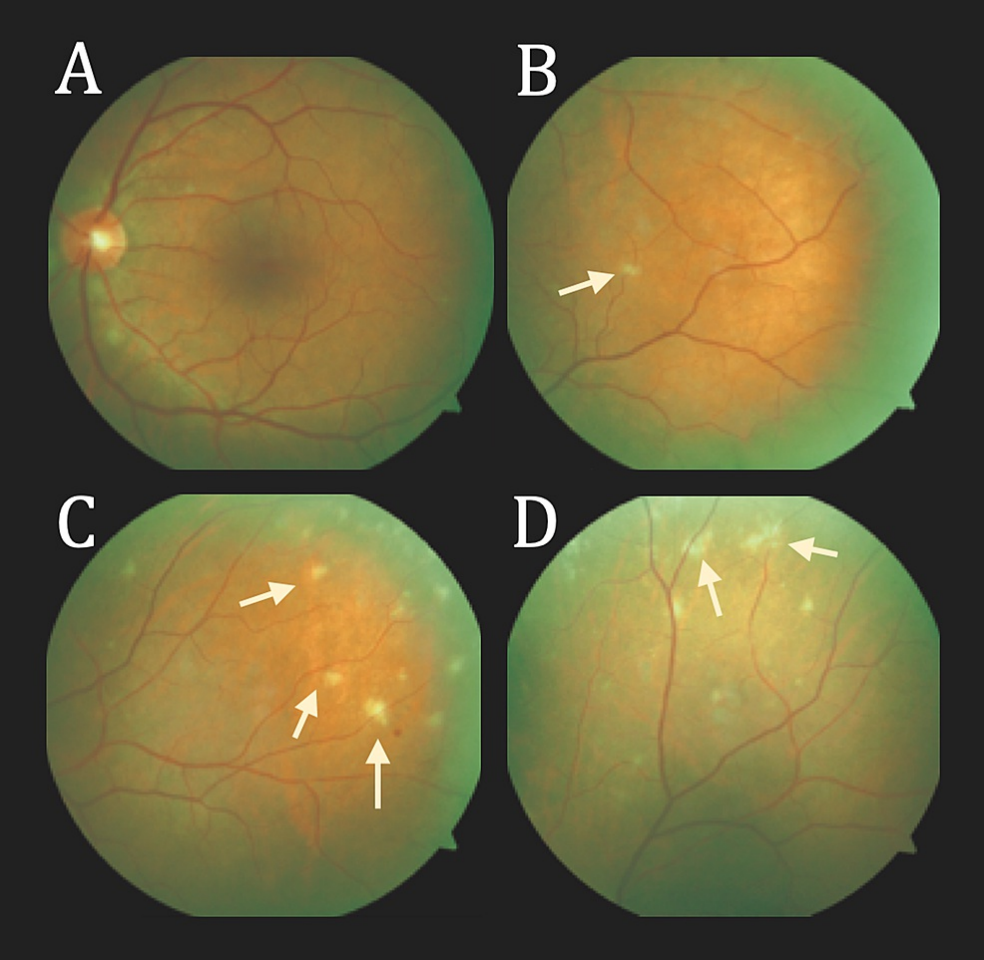
Initial fundoscopy notable for peripheral inflammatory lesions Four conventional 30-degree fundus images of the left eye demonstrating different regions of the retina in the posterior pole. (A) The central retina without major findings. (B-D) Peripheral regions of the retina with yellow-white spots (indicated by the arrows) having the appearance of Dalen-Fuchs nodules.

A 52-year-old Hispanic male presented to the emergency department (ED) with a two-week history of blurry vision and redness in the left eye. The patient’s past medical history was significant for primary VZV infection with associated vitritis in his left eye 23 years prior to presentation, as well as cataract extraction with implant of an intraocular lens in the right eye two weeks prior to presentation. The patient’s initial visual impairment and pain were attributed to presumed sympathetic ophthalmia (SO), secondary to possible trauma during intraocular lens implantation [11]. Initial fundoscopy before presentation to the ED noted peripheral white-yellow lesions, which were presumed to be Dalen-Fuchs nodules, clusters of epithelioid pigment-containing cells indicative of the granulomatous inflammation, and uveitis which is seen in SO (Figures 1A-1D). The patient was subsequently started on high-dose prednisone following a tapering dose. Pain resolved in the left eye, however, blurry vision and redness persisted. The lack of bilateral involvement, worsening symptoms despite corticosteroid treatment, fundus findings, and timing of disease progression suggested ARN as the leading diagnosis as opposed to SO in this patient and oral valacyclovir was subsequently initiated.

**Figure 2:**
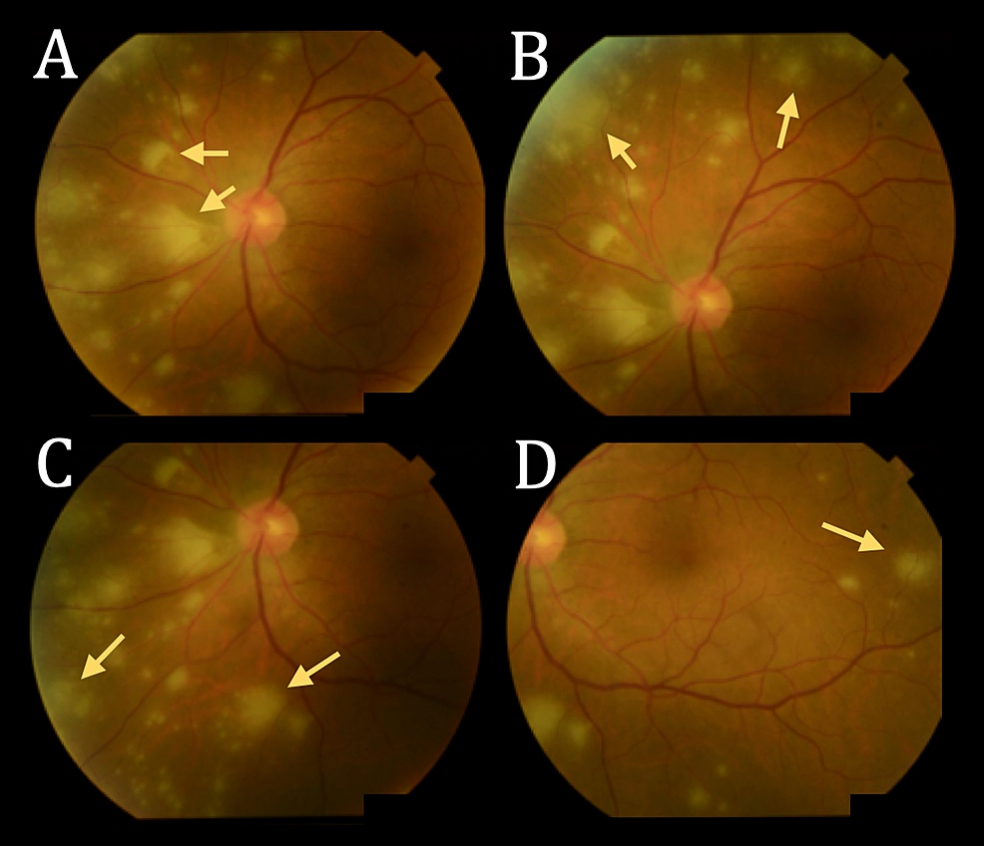
Fundoscopy showing progression of acute retinal necrosis despite treatment with oral acyclovir Four conventional 30-degree fundus images of the left eye in different regions of the posterior pole (A) central; (B) superonasal; (C) inferonasal; (D) temporal. The scattered yellow-white spots (indicated by arrows) represent areas of acute retinal necrosis. Compared to the patient's prior fundoscopy, there is now marked involvement of the central retina and an increased number of lesions in the periphery.

Lack of response to oral valacyclovir prompted the patient to present to the ED one week later. At that time, ocular examination findings indicated decreased visual acuity in the left eye which did not improve with a pinhole occluder, but otherwise normal cranial nerve function. Slit-lamp examination of the left eye suggested an inflammatory reaction, with the presence of white cells in the anterior chamber and keratic precipitates within the cornea. Fundoscopy showed vasculitis with surrounding heme, retinal whitening, and white-yellow multifocal scattered lesions (Figures 2A-2D). Optic nerve head integrity was noted to be intact without hyperemia or edema. The examination of the right eye was normal. Gram stain, viral culture, and bacterial culture of vitreous fluid yielded negative findings. Serology for HIV-1 and HIV-2 were negative, as well as HSV-1, HSV-2, and EBV. However, serology was significant for CMV and VZV IgG antibodies, indicating that the patient had previous exposure to these viral agents. The lack of response to oral valacyclovir warranted the use of intravenous acyclovir.

One day after the presentation, macular lesions extending to the optic disc were noted in addition to worsening symptoms despite IV and oral antiviral agents. Intravitreal injection of ganciclovir was performed given the risk of vision loss, and coverage of CMV as well as traditional ARN agents (HSV, VZV). Four days post-presentation, corneal keratic precipitates resolved, and retinal lesions appeared to have stabilized by bedside fundoscopy (although funduscopy was difficult due to heme). A second dose of intravitreal ganciclovir was given.

**Figure 3:**
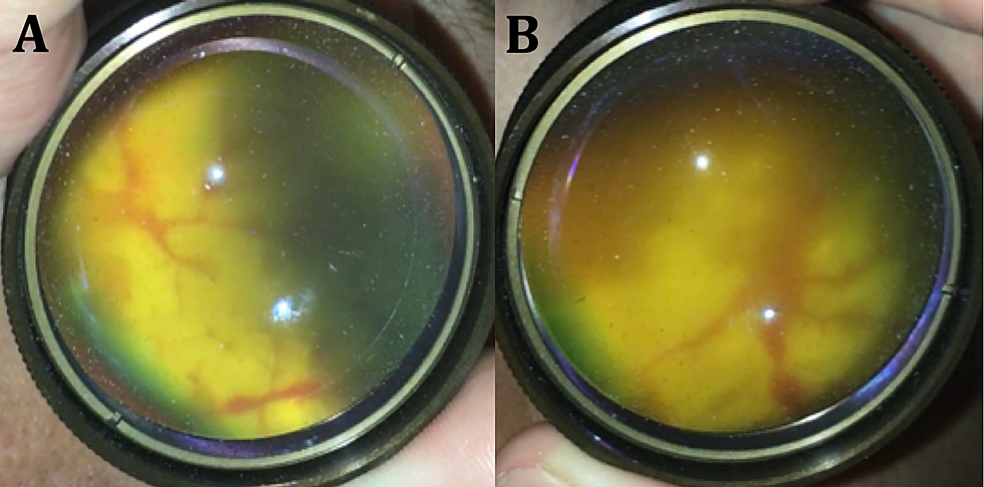
Bedside indirect ophthalmoscopy after three doses of intravitreal ganciclovir Two fundus images from the left eye were captured using a handheld camera and a 20 diopter lens. (A) Posterior pole with superior and nasal aspects in focus. (B) Posterior pole with inferior and temporal regions in focus. No acute inflammatory lesions are visible in A or B; it seems the acute retinal necrosis stabilized after treatment with intravitreal ganciclovir. The red streaks are suggestive of perivascular heme and the blurred dark regions are suggestive of old hemorrhage. The two bright white spots in each image are reflection artifacts from the light source rather than abnormalities of the retina.

**Figure 4:**
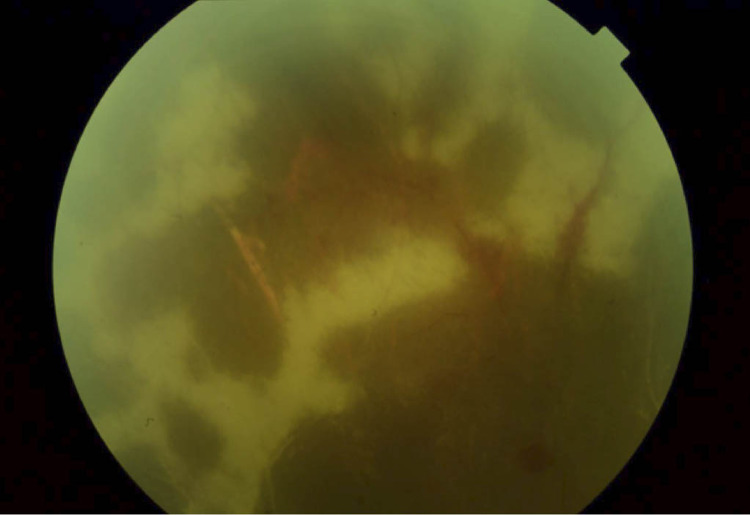
Fundoscopy demonstrating resolution of acute retinal necrosis progression Conventional 30-degree fundus image showing a flat retina, no acute inflammatory lesions, and no signs of active vasculitis. The dark-colored regions are areas of resolved necrosis, old heme, and scarring.

Seven days after hospitalization, bedside fundoscopy demonstrated intact attachment of the retina, decreasing vasculitis, and a decrease in size of the lesions in the nasal area (Figures 3A, 3B). A third intravitreal ganciclovir dose was given and the patient was subsequently discharged and returned one week later for follow-up in the clinic. Fortunately, the patient did not develop any retinal detachment. Fundoscopy of the retina was significant for an infarcted vessel, flattening of lesions, and perivascular heme and overall showed resolution of acute retinal necrosis progression (Figure 4).

## Discussion

Intravitreal injections usually do not serve as the first-line modality for drug delivery to the ocular compartment due to the nature of the procedure and risks for adverse iatrogenic effects, further risk of endophthalmitis, reflux, or pain associated with certain injection techniques [12,13]. In the context of antiviral therapy, oral and intravenous medications have achieved success in patients with acute retinal necrosis, while offering flexibility in dosing, drug delivery, and monitoring by clinicians [14]. However, intravitreal injections remain a mainstay for utilization in the clinical context due to the benefit of maximizing the vitreal concentration of drugs, while mitigating systemic drug toxicity [15]. Here, we report its beneficial use in an immunocompetent patient who did not respond to oral and intravenous antiviral therapy.

Comparing oral to intravenous drug delivery modalities, oral dosing may seem to reach pharmacologically beneficial vitreal concentrations more efficiently at supercritical antiviral doses. However, evidence from retrospective analyses of patients with ARN has not shown a statistically significant difference in corrected visual acuity, long-term severe vision loss, or rates of retinal detachment when comparing oral with intravenous routes of antiviral administration [16]. However, the literature has shown that patients who do not benefit from oral or intravenous antiviral therapy may benefit from the utilization of intravitreal injection, possibly due to direct antiviral drug delivery to the site of pathology, and the level of drug that is delivered to the vitreal compartment [17,18].

One theory for why intravitreal drug delivery shows greater efficacy in complicated cases is that these cases may involve antigen-specific immune deviation, warranting a higher concentration of drug in the affected physiologic compartment [19]. The choice of which antiviral medication to use is another factor clinicians should scrutinize when using an intravitreal antiviral injection. While serology allows clinicians to confirm viral etiologies, sometimes it is necessary to utilize empiric treatment in the setting of acute cases where serology is not readily obtainable due to a delay in laboratory analysis in the institutional setting. An effective clinical history and physical examination allow physicians to make reasonable conjectures while serology is being obtained. Most cases of ARN are due to HSV-1 and HSV-2, which can be treated effectively with acyclovir and foscarnet. Foscarnet also produces extended CMV, VZV, and HIV coverage, and may be beneficial in ARN patients with a history of HIV or low cluster of differentiation 4+ (CD4+) count. However, it should be noted that HIV status might expose a patient to previously aforementioned viral necrosis due to immunosuppression, thus targeted therapy based on serology or PCR results should be the gold standard for therapy [14]. An adverse effect of intravitreous foscarnet injection is the crystallization of the medication in the vitreous, which may or may not damage the retina depending on the configuration of the crystals [20]. Ganciclovir, which is effective against CMV ARN, was utilized in this patient due to the presence of CMV IgM antibodies and the relative convenience of acquiring ganciclovir at the specific institution where the patient was treated.

## Conclusions

This report highlights the utility of the intravitreal route of administration of antivirals in ARN, especially in the less common situation of the local vitreoretinal disease in immunocompetent patients. Based on the findings of this case, we suggest that intravitreous ganciclovir could be considered as the first-line therapy (or as an adjunct to be given simultaneously with intravenous antivirals) for immunocompetent patients presenting with severe unilateral ARN.
